# Positional Cloning of a Type 2 Diabetes Quantitative Trait Locus; *Tomosyn-2*, a Negative Regulator of Insulin Secretion

**DOI:** 10.1371/journal.pgen.1002323

**Published:** 2011-10-06

**Authors:** Sushant Bhatnagar, Angie T. Oler, Mary E. Rabaglia, Donald S. Stapleton, Kathryn L. Schueler, Nathan A. Truchan, Sara L. Worzella, Jonathan P. Stoehr, Susanne M. Clee, Brian S. Yandell, Mark P. Keller, Debbie C. Thurmond, Alan D. Attie

**Affiliations:** 1 Department of Biochemistry, University of Wisconsin-Madison, Madison, Wisconsin, United States of America; 2 Department of Cellular and Physiological Sciences, University of British Columbia, Vancouver, Canada; 3 Department of Statistics, University of Wisconsin-Madison, Madison, Wisconsin, United States of America; 4 Department of Pediatrics, Indiana University School of Medicine, Indianapolis, Indiana, United States of America; Columbia University, United States of America

## Abstract

We previously mapped a type 2 diabetes (T2D) locus on chromosome 16 (Chr 16) in an F2 intercross from the BTBR T (+) tf (BTBR) *Lep^ob/ob^* and C57BL/6 (B6) *Lep^ob/ob^* mouse strains. Introgression of BTBR Chr 16 into B6 mice resulted in a consomic mouse with reduced fasting plasma insulin and elevated glucose levels. We derived a panel of sub-congenic mice and narrowed the diabetes susceptibility locus to a 1.6 Mb region. Introgression of this 1.6 Mb fragment of the BTBR Chr 16 into lean B6 mice (B6.16^BT36–38^) replicated the phenotypes of the consomic mice. Pancreatic islets from the B6.16^BT36–38^ mice were defective in the second phase of the insulin secretion, suggesting that the 1.6 Mb region encodes a regulator of insulin secretion. Within this region, syntaxin-binding protein 5-like (*Stxbp5l*) or tomosyn-2 was the only gene with an expression difference and a non-synonymous coding single nucleotide polymorphism (SNP) between the B6 and BTBR alleles. Overexpression of the b-tomosyn-2 isoform in the pancreatic β-cell line, INS1 (832/13), resulted in an inhibition of insulin secretion in response to 3 mM 8-bromo cAMP at 7 mM glucose. In vitro binding experiments showed that tomosyn-2 binds recombinant syntaxin-1A and syntaxin-4, key proteins that are involved in insulin secretion via formation of the SNARE complex. The B6 form of tomosyn-2 is more susceptible to proteasomal degradation than the BTBR form, establishing a functional role for the coding SNP in tomosyn-2. We conclude that tomosyn-2 is the major gene responsible for the T2D Chr 16 quantitative trait locus (QTL) we mapped in our mouse cross. Our findings suggest that tomosyn-2 is a key negative regulator of insulin secretion.

## Author Summary

Humans carry many genetic variants that confer small effects on metabolic traits relevant to type 2 diabetes. These effects are amplified by environmental stressors like obesity. We used morbid obesity as a sensitizer to identify genes that contribute to the diabetes susceptibility of the BTBR mouse strain. Using mapping and breeding strategies, we were able to narrow a genetic region to one containing just 13 genes. One of these genes, tomosyn-2, emerged as a prime candidate. Our functional studies showed that tomosyn-2 is an inhibitor of insulin secretion, and it binds to the proteins involved in the fusion of insulin containing granules with the plasma membrane. We found a coding mutation and demonstrated that this mutation affects the stability of the protein product. Our work with Tomosyn-2 provides new insights into the regulation of insulin secretion and emphasizes that negative regulation is critical for avoiding insulin-induced hypoglycemia.

## Introduction

Genetic factors are estimated to contribute approximately 50% towards the risk of developing type 2 diabetes (T2D) [1]. Recent genome-wide association studies have identified a number of “diabetes genes”, gene loci that act in an additive manner and conspire with obesity to augment the risk of T2D. Nearly all of these genes affect β-cell function and/or the maintenance of β-cell mass. Thus, it appears that diet and obesity place demands on β-cells for insulin by causing insulin resistance, but the genetic bottleneck that can lead to T2D involves genes that affect the capacity of pancreatic β-cells to meet the increased insulin demand.

Mouse intercrosses provide high power to detect linkage of gene loci to physiological traits related to obesity and diabetes. The mapping resolution of these intercrosses is typically not fine enough to identify individual genes. However, by generating panels of congenic strains carrying meiotic recombinations within disease loci, it is possible to map the genes underlying those loci with high resolution. We recently reported the positional cloning of a T2D gene, *Sorcs1*, to sub-genetic resolution using this approach [2]. Several other genes have been identified using a similar approach; *Zfp69,* which encodes a transcription factor involved in regulating glucose levels, was identified as the gene underlying a diabetes susceptibility locus on mouse chromosome 4 [3]. Similarly, *Lisch-like* was identified as the gene underlying a T2D locus on chromosome 1 and was shown to be involved in regulating β-cell mass and plasma glucose levels [4].

Borrowing from microbial genetics, mouse genetic studies employ a powerful tool for increasing the sensitivity to detect heritable phenotypes. This involves sensitized screens wherein a severe stressor provokes phenotypes that would otherwise be silent. The stressor need not be a normal feature in human disease pathogenesis to evoke phenotypes of great relevance to disease. For example, the apoE-deficient mouse is the most widely used animal model of atherosclerosis even though apoE deficiency is extremely rare in humans [5]. Similarly, a mutation in the Leptin gene (*Lep^ob^*) promotes morbid obesity in mice (*Lep^ob/ob^*) and evokes dysregulation of many pathways, enabling a greater understanding of their regulatory mechanisms [6], [7].

Using the *Lep^ob^* mutation as a stressor, we found that the BTBR T (+) (BTBR) mouse strain develops severe T2D, whereas the C57BL/6 (B6) strain has moderate hyperglycemia and expands its β-cell mass [8], [9]. In an F2 intercross derived from these two strains, we identified a T2D susceptibility locus on chromosome 16 (Chr 16) [9]. In the present study, we developed a panel of congenic strains that enabled us to narrow this locus to just 0.94 Mb. Lean congenic mice that contain this genomic region derived from the BTBR strain have elevated glucose and reduced insulin levels. Islets from these mice show deficiencies in insulin secretion. Within this small interval, we identified a novel diabetes susceptibility gene, *Syntaxin binding protein 5 like* (*Stxbp5l*), also known as *Tomosyn-2.* We showed that the tomosyn-2 protein is an inhibitor of insulin secretion.

## Results

### Chr 16 consomic mice have increased fasting plasma glucose and reduced insulin levels

**Figure 1:**
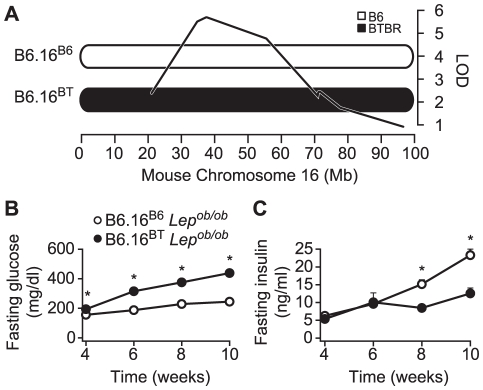
Chromosome 16 of BTBR mice contains diabetogenic alleles. A) Consomic mice were generated by introgression of either a B6 or BTBR mouse chromosome 16 in the B6 *Lep^ob/ob^* mice. The LOD curve corresponds to a fasting glucose QTL from an F2 cross of (B6 x BTBR) *Lep^ob/ob^* mice. The approximate QTL position was derived using a previously described method [9]. Fasting plasma glucose (B) and fasting plasma insulin (C) were measured after a 4 h fast in male B6.16^B6^ and B6.16^BT^ *Lep^ob/ob^* mice. Values are means ± S.E. of N≥34. * *p*≤0.05 for B6.16^BT^ *Lep^ob/ob^* mice vs. control B6.16^B6^ mice at each time point.

We previously identified a fasting plasma glucose locus on Chr 16 from a *Lep^ob/ob^* F2 intercross derived from the B6 and BTBR mouse strains [9]. This locus acts in a fully dominant fashion on plasma glucose and a semi-dominant fashion on fasting plasma insulin [9]. The LOD peak on Chr 16 of the fasting glucose locus from the F2 intercross is located at approximately 36–38 Mb (Figure 1A). To determine if the Chr 16 locus could act autonomously to affect T2D susceptibility, we derived a chromosome substitution (i.e. consomic) mouse strain by introgression of Chr 16 from BTBR into B6 *ob/ob* mice (B6.16^BT^ *Lep^ob/ob^*). The fasting plasma glucose levels of the resulting consomic mice were significantly elevated at 4, 6, 8, and 10 weeks compared to control (B6.16^B6^ *Lep^ob/ob^*) mice and accounted for a major proportion of the plasma glucose phenotype of the parental BTBR strain. Fasting plasma insulin levels were reduced at 8 and 10-weeks in B6.16^BT^ *Lep^ob/ob^* mice compared to B6.16^B6^ *Lep^ob/ob^* mice (Figure 1B and 1C). These data suggested that the hyperglycemia caused by BTBR Chr 16 substitution is due to reduction in insulin levels. The data also indicate that the locus on Chr 16 acts autonomously (i.e. in the absence of BTBR alleles on other chromosomes) to affect glucose and insulin levels.

### Congenic mice with a 1.6 Mb fragment of the BTBR Chr 16 have an insulin secretion defect

**Figure 2:**
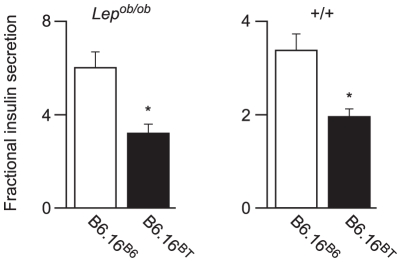
Effect of substitution of BTBR chromosome 16 in the B6 *Lep^ob/ob^* mice on insulin secretion. Islets were isolated from *Lep^ob/ob^* (left graph) and lean (right graph) 10-week B6.16^B6^ and B6.16^BT^ male mice. *Lep^ob/ob^* islets were incubated with 16.7 mM glucose and the islets from the lean mice were incubated with 3 mM 8-bromo cAMP at 11.1 mM sub-maximal glucose. Values are mean ± S.E. of N≥3. *p≤0.05 for fractional insulin secretion from islets of B6.16^BT^ mice vs. control B6.16^B6^ mice.

To assess whether the B6.16^BT^ *Lep^ob/ob^* mice have a defect in insulin secretion, we isolated pancreatic islets from 10-week old B6.16^BT^ *Lep^ob/ob^* mice and measured fractional insulin secretion in response to high glucose (16.7 mM). We observed a ∼50% reduction in fractional insulin secretion in the B6.16^BT^ *Lep^ob/ob^* islets relative to control mice (B6.16^B6^ *Lep^ob/ob^*) (Figure 2, left graph).

To avoid the metabolic complexities that are attributed to the leptin mutation in the *Lep^ob/ob^* mice [10], we performed experiments in lean mice. Islets isolated from the congenic B6.16^BT^ and B6.16^B6^ lean mice were treated with 8-bromo cAMP (3 mM) at sub-maximal glucose (11.1 mM); this combination of secretagogues was used for phenotyping lean congenic mice because it evoked more insulin secretion from lean islets than glucose alone. We observed ∼40% reduction in fractional insulin secretion in islets isolated from the lean B6.16^BT^ mice relative to the lean control B6.16^B6^ mice (Figure 2, right graph). The data show that the insulin secretion defect, although initially mapped in a screen of F2 mice sensitized by the *Lep^ob^* mutation, manifests itself independent of leptin deficiency.

**Figure 3:**
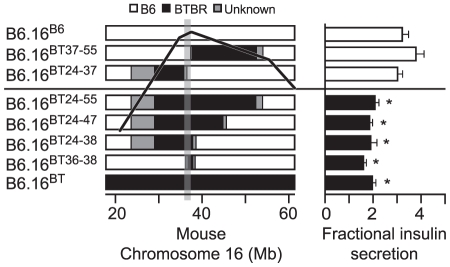
Effect on insulin secretion of introgressing 1.6 Mb of BTBR Chr 16 into B6 mice. Left panel: Congenic mice were generated by introgression of varying fragments of the BTBR chromosome 16 into the B6 mice. The B6/BTBR boundaries for each congenic strain were determined via microsatellite marker, SNP sequencing or DIP sequencing and are listed in Datasets S1 and S3. Overlaying the congenic diagram is the position of the fasting glucose QTL derived from a (B6 x BTBR) *Lep^ob/ob^* F2 intercross. Right panel: Fractional insulin secretion (% of islet insulin content) from the islets of the 10-week lean congenic mice. Isolated islets were incubated for 45 min in KRB-based buffer containing low glucose (1.7 mM). After 45 min, the islets were exposed to 8-bromo cAMP (3 mM) at sub-maximal glucose (11.1 mM) for another 45 min. Values are mean ± S.E. of N≥3. *p≤0.05 for fractional insulin secretion from islets of congenic mice that contains the 1.6 Mb fragment of the BTBR chromosome 16 mice vs. congenic mice carrying this 1.6 Mb fragment derived from the B6 strain.

To investigate the region of the BTBR Chr 16 that confers the insulin secretion defect, a panel of lean congenic mouse strains was generated from the B6.16^BT^ mice, each containing a small introgressed region from the BTBR Chr 16 in the B6 background (Figure 3, left panel). The B6/BTBR boundaries for each congenic strain were determined via microsatellite marker, single nucleotide polymorphism (SNP) sequencing or deletion/insertion polymorphism (DIP) sequencing (Dataset S1). By phenotyping each strain, we were able to fine-map the location of the gene responsible for the insulin secretion defect.

Islets were isolated from each lean congenic mouse strain and fractional insulin secretion was determined in the presence of 3 mM 8-bromo cAMP at sub-maximal 11 mM glucose. The islets isolated from the lean congenic mice that carry the 2 Mb Chr 16 derived from the BTBR strain (B6.16^BT36–38^) were defective in insulin secretion when compared to islets isolated from congenic mice B6.16^B6^, B6.16^BT37–55^, or B6.16^BT24–37^ (Figure 3, right panel). The overlapping region of BTBR Chr 16 represented by congenic strains, B6.16 ^BT36–38^ and B6.16 ^BT37–55^ enabled us to further narrow the region to 1.6 Mb. Islets isolated from congenic mice, B6.16^BT24–55^, B6.16^BT24–47^, or B6.16^BT24–38^, which contained the 1.6 Mb region derived from the BTBR strain, also displayed a reduced level of insulin secretion, indicating that this 1.6 Mb region contains a gene responsible for regulating insulin secretion.

### The 1.6 Mb region of the BTBR Chr 16 is sufficient to increase plasma glucose levels

**Figure 4:**
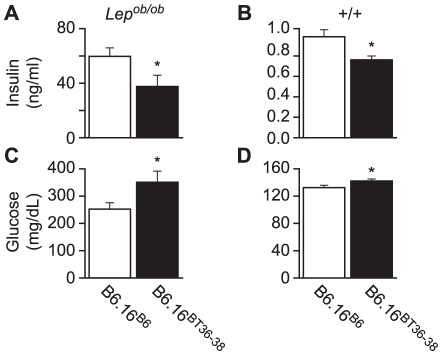
Plasma insulin and glucose levels in the B6.16^BT36–38^ male mice. Random-fed plasma insulin (A and B) and glucose (C and D) were measured in 10-week *Lep^ob/ob^* and lean B6.16^B6^ and B6.16^BT36–38^ male mice. Values are means ± S.E. of N≥10. *p≤0.05 for plasma glucose and insulin levels in B6.16^BT36–38^ mice vs. control B6.16^B6^ mice.

We next determined the effect of introgression of the BT36–38 region of Chr 16 into B6 mice on susceptibility to obesity-induced diabetes. Plasma insulin and glucose levels were determined in random-fed 10-week B6.16^BT36–38^ and control B6.16^B6^ *Lep^ob/ob^* congenic mice. We observed a ∼40% reduction in plasma insulin levels in the B6.16^BT36–38^ *Lep^ob/ob^* compared to B6.16^B6^ *Lep^ob/ob^* mice (Figure 4A). The reduction in plasma insulin was accompanied by an increase in plasma glucose by ∼100 mg/dL (Figure 4C).

Although not nearly as dramatic as in the *Lep^ob/ob^* mice, lean congenic mice with the 36–38 Mb BTBR insert had a significant reduction in plasma insulin and increase in plasma glucose (Figure 4B and 4D, respectively). Detection of this very small rise in plasma glucose required a very large sample size (n = 80) to achieve statistical significance. Clearly, we would not have found this modest phenotype in a screen of lean mice, showing that severe stressors like the *Lep^ob^* mutation are required to identify subtle allelic variation in QTLs that contribute to T2D risk.

### Reduced insulin secretion in islets from lean B6.16^BT36–38^ mice

Insulin secretion from pancreatic β-cells is biphasic. The first phase represents a brief but rapid secretion from pre-docked insulin granules in response to an initial depolarization of the plasma membrane. Non-nutrient secretagogues like KCl predominantly invoke the first phase of insulin secretion. The second phase of insulin secretion is associated with metabolic signals derived from the metabolism of fuel-based insulin secretagogues like glucose. Glucose affects both the first and second phase of insulin secretion. Briefly, glucose oxidation increases the ATP/ADP ratio, resulting in the closure of ATP-sensitive K_ATP_ channels. This causes depolarization of the plasma membrane and influx of Ca^2+^ via L-type voltage-dependent calcium channels. Glucose promotes the second phase of insulin secretion without causing a further increase in intracellular Ca^2+^ levels. Diazoxide inhibits closure of the K^+^ channels, therefore adding this drug along with 16.7 mM glucose will result only in the glucose-induced second phase of insulin secretion and eliminates any glucose induction of the first phase of insulin secretion [11]–[13].

To determine which phase of insulin secretion is defective in our lean congenic B6.16^BT36–38^ mice, we carried out perifusion studies of isolated islets. Islets were perifused in Krebs-Henseleit Ringer bicarbonate (KRB) buffer at the rate of 1 ml/min. The perfusate was sampled every 30 sec, and the secreted insulin was measured by ELISA. After an initial 60 min equilibration period in KRB containing 1.7 mM glucose, islets were perifused for 10 min in KRB containing 40 mM KCl and 250 µM diazoxide to elicit first phase of insulin secretion. After 10 min, the islets were perifused for an additional 30 min in KRB containing 16.7 mM glucose with 40 mM KCl and 250 µM diazoxide to evoke the second phase of insulin secretion. The peak of the first phase of insulin secretion from B6.16^B6^ islets was observed within 1–2 min of KCl treatment. Following the first peak, the more sustained second phase of insulin secretion was observed for an additional 30 min, mimicking the well-studied biphasic kinetics of insulin secretion [14].

**Figure 5:**
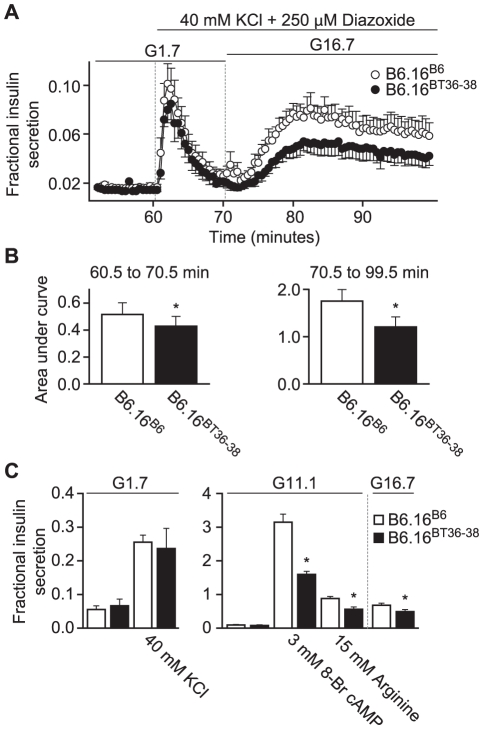
Impaired insulin secretion in islets isolated from the B6.16^BT36–38^ mice. Islets were isolated from 10-week male control B6.16^B6^ mice (open circles) or B6.16^BT36–38^ mice (filled circles). A) Islets were preincubated for 60 min in low glucose (1.7 mM) and basal insulin release was determined to establish a base line. After 60 min, the buffer was changed to high potassium (40 mM) for another 10 min to obtain first-phase insulin secretion. The glucose concentration was then increased to 16.7 mM for 30 min to obtain second phase insulin. Diazoxide (250 mM) was present in high potassium (40 mM) and high glucose (16.7 mM) buffer. Eluted fractions containing secreted insulin were collected and insulin concentration was determined by ELISA. B) The area under the curve (AUC) was determined for the first (60.5–70.5 min, left graph) and second phase (70.5–99.5 min, right graph) of insulin secretion for the B6.16^BT36–38^ and B6.16^B6^ mice. Values are means ± S.E. of N = 5. *p≤0.05 for the first or second phase insulin secretion from islets isolated from the B6.16^BT36–38^ mice vs. control B6.16^B6^ mice. C) Static insulin secretion studies. Isolated islets were pre-incubated for 45 min in low glucose (1.7 mM) buffer. After 45 min, the islets were incubated in the presence of various insulin secretagogues. Values are means ± S.E. of N≥4. *p≤0.05 for static insulin secretion from islets isolated from the B6.16^BT36–38^ mice vs. control B6.16^B6^ mice. KCl = potassium chloride.

Islets from B6.16^BT36–38^ lean mice secreted ∼40% less insulin during the second phase than islets from the B6.16^ B6^ mice, as determined by calculating the area under the curve (AUC) (Figure 5A, 5B). There was also a small, but statistically significant reduction of first-phase insulin secretion (*p* = 0.044) (Figure 5A, 5B).

**Figure 6:**
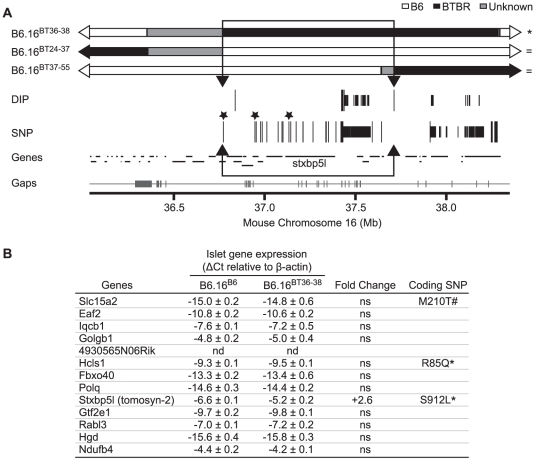
Insulin secretion defective region narrowed to 0.94 Mb on mouse chromosome 16 containing 13 genes. (A) Results from Agilent SureSelect Target Enrichment of a 35.35 to 38.65 Mb region on mouse chromosome 16 followed by Next Generation Sequencing. Listed are single nucleotide polymorphisms (SNP) and deletion insertion polymorphisms (DIP) between B6 and BTBR (listed in Dataset S2). Top: congenic maps (with respective B6, BTBR, and unknown domains – boundaries were determined via SNP/DIP sequencing analysis listed in Dataset S1) of the strains used to further define the region linked to the insulin secretion defect. Strains are listed as defective (*) or normal for insulin secretion ( = ). Those SNPs and DIPs subjected to additional confirmation are represented as longer lines. Non-synonymous coding SNPs are highlighted with asterisks. The boundaries of the inferred QTL region were defined by 1) the phenotypes of the sub-congenic strains and 2) sequencing of the region. We were able to exclude the grey region common to B6.16^BT36–38^ and B6.16^BT24–37^ because 1) the latter strain is phenotypically like B6 and the former strain is phenotypically like BTBR and 2) sequencing showed that the region is identical by descent between B6 and BTBR. Bottom: genes located in this region as well as the breadth of the sequence coverage area (with gaps shown as tick marks along the line) (Gaps are listed in Dataset S4). The 0.94 Mb region containing the locus for defective insulin secretion is represented as an open box with arrowheads. B) Genes in the 0.94 Mb region with their measured islet gene expression, fold change, and presence of non-synonymous coding SNPs. Not determined (nd), not significant (ns), * SNP published in Mouse Phenome Database and UCSC Genome Browser, # SNP published only in UCSC Genome Browser.

To complement the perifusion experiments, static insulin secretion experiments were performed in islets isolated from the B6.16^BT36–38^ and control B6.16^B6^ lean mice. Isolated islets were incubated for 45 min in KRB containing 1.7 mM glucose. Following a 45-min incubation, the islets were treated with 40 mM KCl in KRB containing 1.7 mM glucose. No difference in fractional insulin secretion was observed in response to KCl (Figure 6C, left panel). However, we observed a significant decrease in fractional insulin secretion between islets from the B6.16^BT36–38^ lean mice and those from the control B6.16^B6^ lean mice in response to 15 mM arginine, 3 mM 8-bromo cAMP in KRB containing 11 mM glucose, and 16.7 mM glucose alone (Figure 5C, middle and right panels).

### Sequencing and alignment of overlapping congenic mouse strains narrowed the Chr 16 locus to 0.94 Mb

To further narrow the 1.6 Mb region of BTBR Chr 16 responsible for the phenotype, we used Agilent's SureSelect Target Enrichment to capture DNA from 35.35 Mb to 38.65 Mb on mouse Chr 16. 55,336 RNA baits were designed using the Agilent eArray and were used to enrich for our region from tail DNA of the B6, BTBR, B6.16^BT36–38^, B6.16^BT24–37^and B6.16^BT24–38^ mice. DNA was sequenced by Next Generation Sequencing using an Illumina GA IIx sequencer at the UW-Madison Biotechnology Center. Using CLC Genomics 4.0.3 Software, we were able to identify 470 SNPs; 3 non-synonymous coding SNPs and 46 DIPs between the B6 and BTBR DNA (Figure 6A). 83 SNPs and 8 DIPs were further confirmed by manual base reading to confirm the accuracy of the software (listed in Dataset S2). Using this sequence and known overlapping regions derived from the BTBR strain in the sub-congenic strains exhibiting normal insulin secretion (B6.16^BT24–37^, B6.16^BT37–55^), we were able to narrow the region responsible for the insulin secretion defect to 0.94 Mb containing 13 genes (Figure 6B).

**Figure 7:**
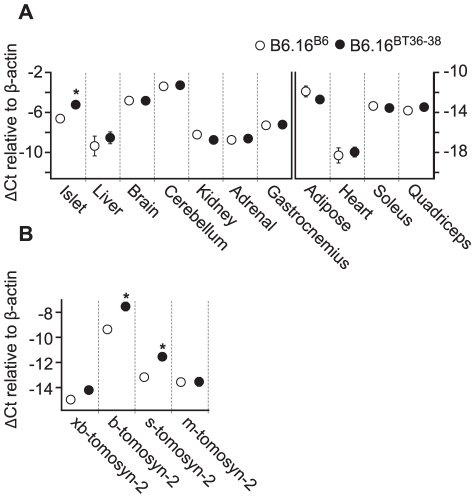
Increased expression of tomosyn-2 gene in islets of the B6.16^BT36–38^ mice. Total mRNA was extracted from islets, liver, brain, cerebellum, kidney, gastrocnemius, adipose, heart, soleus, and quadriceps of the B6.16^BT36–38^ and B6.16^B6^ mice. Relative abundance was determined by real-time PCR of the cDNA. The ΔCt was calculated by subtracting the raw Ct of tomosyn-2 gene from the raw Ct of the β-actin gene. A) The mRNA abundance of the tomosyn-2 gene in different tissues. Left: tissues that have a relatively higher level of tomosyn-2 expression. Right: tissues with a relatively low level of tomosyn-2 expression. B) Relative expression of the tomosyn-2 isoforms in the islets. Values are means ± S.E. of N≥4. *(*p*≤0.05 for the expression of tomosyn-2 gene in tissues of the B6.16^BT36–38^ mice vs. control B6.16^B6^ mice.

To identify the gene(s) responsible for the insulin secretion defect, each candidate gene in the 0.94 Mb region was scored for the difference in mRNA abundance between the islets B6.16^B6^ and B6.16^BT36–38^ islets, the presence of non-synonymous coding SNPs, and similarity to a protein that have a functional role in exocytosis. *Tomosyn-2* or *Stxbp5l (syntaxin binding protein 5-like)* quickly emerged as the top candidate gene. The mRNA abundance of *tomosyn-2* was elevated 2.6 fold in the B6.16^BT36–38^ lean mice compared to control B6.16^B6^ mice (Figure 7A). *Tomosyn-2* has a coding SNP (Ser-912→Leu). Eight other SNPs were also identified in the introns and additional SNPs were identified in the intergenic regions 5′ and 3′ of the gene (Dataset S1, Table S1). The tomosyn-2 protein shares 95% identity in the C-terminal soluble NSF (N-ethylmaleimide-sensitive factor) attachment protein receptor (SNARE) domain with several syntaxin-binding proteins. Finally, a related protein, tomosyn-1, has been shown to inhibit insulin secretion [15].

### Increased expression of tomosyn-2 in islets of the B6.16^BT36–38^ lean mice

To understand the role of tomosyn-2 in the regulation of insulin secretion, the expression of tomosyn-2 was determined in key metabolic tissues; islet, liver, brain, cerebellum, kidney, adrenal, adipose (perigonadal), heart, skeletal muscle (gastrocnemius, soleus, and quadriceps) of the lean B6.16^BT36–38^ and B6.16^B6^ mice. The mRNA expression of tomosyn-2 in islets of the lean B6.16^BT36–38^ mice was ∼2.6-fold higher than in islets from the B6.16^B6^ mice (Figure 7A). No allele-dependent difference in the tomosyn-2 expression was observed in liver, brain, cerebellum, kidney, adrenal, gastrocnemius, adipose, heart, soleus, and quadriceps between the lean B6.16^BT36–38^ and B6.16^B6^ mice.

Four tomosyn-2 isoforms have been identified in mice: xb-, b, s, and m-tomosyn-2. We determined the relative expression of the tomosyn-2 isoforms in islets of the lean B6.16^BT36–38^ and B6.16^B6^ mice. We found that the *b-tomosyn-2* isoform is the most abundant isoform in mouse islets. This was confirmed by RT-PCR with a primer pair that simultaneously amplified all of the tomosyn-2 isoforms (data not shown). The relative expression of *b-tomosyn-2* and *s-tomosyn-2* mRNA was ∼6-fold higher in islets of the lean B6.16^BT36–38^ mice than in lean B6.16^B6^ mice (Figure 7B). We observed no significant difference between the two-congenic mouse strains in the expression of *xb-* and *m-tomosyn-2* isoforms. Together, the data indicate that increased expression of *tomosyn-2* may be responsible for the insulin secretion defect observed in the lean B6.16^BT36–38^ mice.

### Tomosyn-2 inhibits insulin secretion in pancreatic β-cells

**Figure 8:**
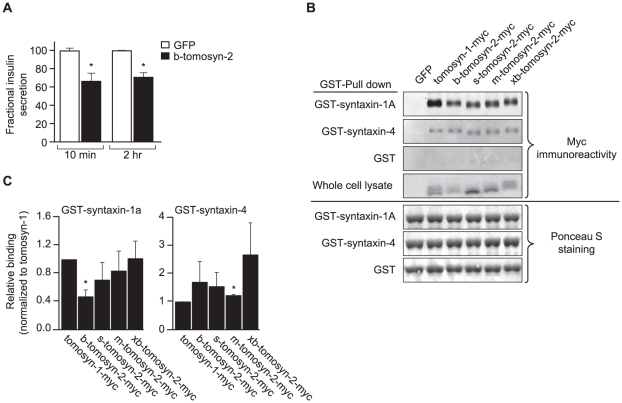
Overexpression of tomosyn-2 inhibits insulin secretion in INS1 (832/13) cells and binds syntaxin-1A and -4. A) INS1 (832/13) cells were plated in a 96-well plate (10^5^ cells/well). After overnight incubation, the cells were cultured in RPMI supplemental media and transfected with a bicistronic mammalian expression plasmid containing either GFP or b-tomosyn-2 and GFP. After 36 h of incubation, the insulin secretion was measured. Fractional insulin secretion in response to 3 mM 8-bromo cAMP at 7 mM glucose from b-tomosyn-2 transfected cells was normalized to that of cells transfected with GFP. Values are means ± S.E. of N≥4. * *p*≤0.05 for the fractional insulin secretion from the cells overexpressing tomosyn-2 vs. GFP expressing cells. B) HEK 293T cells were transfected with mock, tomosyn-1-myc, or tomosyn-2-myc isoforms (b, s, m, and xb) mammalian expression plasmids. After 24 h, the cells were harvested and whole cell lysates (WCL) were prepared. GST-pull-down experiments were performed as described in Methods. Ponceau S staining shows input of the GST fusion proteins. The overexpression of the exogenous protein in HEK 293T cells was determined by subjecting the WCL to western blot using anti-myc antibody. C) The graphs show the binding of each tomosyn-2 isoform relative to the amount of bound tomosyn-1. Values are means ± S.E. of N = 4.

To investigate the role of tomosyn-2 in insulin secretion, we investigated the effect of overexpressing b-tomosyn-2 in the pancreatic β-cell line, INS1 (832/13). The cells were transfected with GFP or b-tomosyn-2 expression plasmids. After 36 h, the cells were incubated in KRB containing 1.5 mM glucose for 2 h. Following the low glucose incubation, the cells were incubated for additional 10 min or 2 h in 3 mM 8-bromo cAMP at 7 mM glucose. Overexpressing b-tomosyn-2 decreased insulin secretion by ∼40% vs. GFP expressing cells at both 10 min and 2 h (Figure 8A). No inhibition in fractional insulin secretion was observed at low glucose (1.5 mM) (data not shown).

To determine if b-tomosyn-2 binds to syntaxin-1A and syntaxin-4, key t-SNARE proteins involved in the fusion of insulin granules to the plasma membrane, in vitro binding experiments were performed using GST fused syntaxin-1A and syntaxin-4 recombinant proteins (soluble, lacking transmembrane domains) by pull-down assays using glutathione beads. All isoforms of tomosyn-2 bound to GST-syntaxin-1A and GST-syntaxin-4 (Figure 8B). The quantitation for the amount of bound tomosyn-2 isoforms as a fraction of total is shown in Figure 8C. Tomosyn-1 was used as a positive control for binding. The GST tag did not pull down tomosyn-1 or tomosyn-2, confirming that the interaction between tomosyn-2 and syntaixn-1A and -4 is specific. Together, these data suggests that the mechanism by which b-tomosyn-2 inhibits insulin secretion involves binding to the syntaxin proteins. This suggests the possibility that tomosyn-2, like tomosyn-1, inhibits insulin secretion by preventing the binding of VAMP2 to syntaxin-1A and syntaxin-4.

### The B6 allelic product of B-tomosyn-2 is susceptible to proteasomal degradation

**Figure 9:**
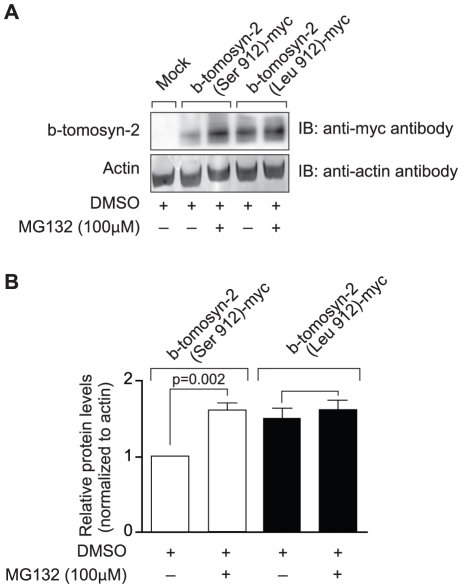
Effect of proteasomal inhibitor on B6 and BTBR allele of b-tomosyn-2. A) The mammalian expression plasmid containing the mock, b-tomosyn-2 (Serine912)-myc or b-tomosyn-2 (Leucine-912)-myc were transfected in HEK293Tcells. After 16 h incubation in DMEM supplemental media, cells expressing b-tomosyn-2-myc were treated with MG132 (100 µM) or DMSO for 6 h. Equal amounts of protein were subjected to western blot analysis and the level of b-tomosyn-2-myc protein was determined using anti-myc antibody. The actin was used as a loading control. B) Shows the quantitation of the signals for three experiments. Values are means ± S.E. of N = 3. *p≤0.05 for the expression of b-tomosyn-2-myc in the samples treated with MG132 vs. DMSO.

We have shown that tomosyn-2 is a negative regulator of insulin secretion and also binds to syntaxin-1A and syntaxin-4. To investigate the possibility that the serine-912leucine SNP in tomosyn-2 affects its stability, HEK293T cells were transfected with empty vector (mock), b-tomosyn-2 (Serine-912), or b-tomosyn-2 (Leucine-912). After 16 h, the cells were treated with or without the proteasomal inhibitor, MG132 (100 µM) for 6 h. The MG132 treatment rescued the B6 allelic form of the protein, b-tomosyn-2 (serine-912), from proteasomal degradation by ∼50% (Figure 9A and 9B). However, the BTBR allelic form of the protein, b-tomosyn-2 (leucine-912) was not resistant to MG132 treatment, suggesting that an increased stability of the tomosyn-2 protein might be responsible for the attenuation in insulin secretion from islets of the BTBR mice.

## Discussion

Our pursuit of genes conferring susceptibility to obesity-induced T2D focuses on two mouse strains that differ in diabetes susceptibility. BTBR mice, when made obese with the *Leptin^ob^* mutation, are susceptible to T2D, whereas B6 mice with the same mutation are relatively diabetes resistant [16], [17]. The diabetes susceptibility of the obese BTBR mice has multiple causes, including an insulin secretion defect and a failure to increase β-cell mass. As early as 4 weeks of age, islets from the obese B6, but not BTBR mice have an increase in expression of a module of cell cycle genes whereas the obese BTBR mice fail to induce the expression of this module [16].

Through genetic mapping in an F2 intercross, we identified a strong QTL on Chr 16 wherein the BTBR allele is linked to increased glucose levels. In complex trait genetics, it is often the case that gene loci do not act autonomously, but must act along with specific alleles at other loci to exert their phenotypic effects [18]–[20]. To determine if the Chr 16 locus acts autonomously, we derived a chromosome substitution strain. Substitution of Chr 16 in the B6 strain with Chr 16 from the BTBR strain led to a ∼100 mg/dl increase in glucose and a ∼50% decrease in plasma insulin. This established that a locus on Chr 16 acts autonomously and is sufficient to account for a major part of the diabetes phenotype of the BTBR mouse strain.

QTL mapping in an F2 intercross does not provide the resolution required to identify individual genes. To narrow the interval, we derived a panel of interval-specific congenic strains. The strains were phenotyped on the basis of insulin secretion from isolated islets, a far more robust phenotype than fasting glucose or insulin levels. This enabled us to narrow the position of the QTL to <1 Mb, containing just thirteen genes. Of these thirteen genes, tomosyn-2 was the only gene that had both altered mRNA abundance and a coding SNP (S912L).

Recent studies by Williams et al. show that tomosyn-2 inhibits exocytosis in PC12 cells [21]. Our experiments establish a role for tomosyn-2 in insulin secretion. When we overexpressed tomosyn-2 in the pancreatic β-cell line INS1 (832/13), insulin secretion in response to 8-bromo cAMP at sub-maximal glucose was attenuated. In vitro GST-pull-down experiments showed that tomosyn-2 has the ability to bind t-SNARE proteins, syntaxin-1A and syntaxin-4. Syntaxin-1A is involved in the first phase and syntaxin-4 is involved in regulating both the first and second phase of insulin secretion [22]. These results establish that tomosyn-2, like its homologue, tomosyn-1, inhibits insulin secretion [23]. The fact that allelic variation in tomosyn-2 is sufficient to produce this phenotype suggests that tomosyn-1 cannot compensate for this deficiency, implying that their functions may not completely overlap.

The mRNA abundance of tomosyn-2 was increased in the congenic mouse strains expressing the BTBR allele. It is difficult to determine if this difference in expression level is sufficient to produce the difference in insulin secretion in islets of the two mouse strains because it would require accurately titrating the gene dosage (and the amount of protein product) in a null background.

Sequence analysis revealed a SNP in tomosyn-2 (S912L). We utilized a recent study showing that the proteasome inhibitor MG132 increases the abundance of tomosyn-2 [21]. We found that the S912L SNP abolishes the ability of MG132 to rescue tomosyn-2 from proteasomal degradation, thus establishing a functional role for the S912L SNP. Therefore, the decreased insulin secretion associated with the BTBR allele might be the result of increased stability of the tomosyn-2 protein as a consequence of the SNP at amino acid 912. We also indentified SNPs in the introns and the intergenic regions 5′ and 3′ of the tomosyn-2 gene (Figure 7). The intronic SNPs may regulate the stability of the tomosyn-2 mRNA and the intergenic SNPs might affect the level of transcription of the gene.

To investigate the role of the S912L SNP in pancreatic islets, we conducted perifusion experiments. Our perifusion experiments demonstrated that islets from the B6.16^BT36–38^ congenic mice were defective in the 2^nd^ phase of insulin secretion. Our results suggest that tomosyn-2 is likely to be responsive to metabolic signals. With static incubation experiments, we tested various insulin secretagogues. Islets with the BTBR allele of tomosyn-2 were clearly less responsive to cAMP or arginine at sub-maximal glucose. These secretagogues are involved in both phases of insulin secretion, but the exact mechanisms by which they stimulate the 2^nd^ phase of insulin secretion are not fully understood. The involvement of tomosyn-2 provides a plausible new target for the actions of these secretagogues.

We show that tomosyn-2, similar to tomosyn-1, binds to syntaxin-1A and syntaxin-4 [24]–[27]. Recent studies suggest that the binding to syntaxin is necessary but not sufficient for tomosyn-2′s inhibition of insulin secretion [21].

Our studies suggest that tomosyn-2 imposes a critical brake on insulin secretion. This is particularly important during fasting when inappropriate insulin secretion could cause life-threatening hypoglycemia. We hypothesize that under fasting conditions when glucose levels are low, tomosyn-2 blocks exocytosis and prevents hypoglycemia.

In mice, the two tomosyn genes, tomosyn-1 and tomosyn-2, encode seven alternatively spliced variants [23]. Tomosyn-1 contains three distinct isoforms (s, m, and b), whereas tomosyn-2 has four different spliced variants (s, m, b, and xb). The spliced exons encode the hypervariable region (HVR), which in tomosyn-1 has been shown to be subject to SUMOylation and PKA-mediated phosphorylation [21], [28]. The amino acid sequences of tomosyn-1 and tomosyn-2 are quite similar in the N-terminal WD40 repeats and C-terminal VAMP-like domain (VLD) [23]. Tomosyn-1 was identified in neurons as a syntaxin-1-binding protein that sequesters t-SNAREs on the plasma membrane by forming a “dead end”, nonfusogenic SNARE complex, resulting in inhibition of the formation of the SNARE complex [24], [26]. Deletion of the tomosyn-1 gene in *C.elegans* or in mice resulted in enhanced asynchronous neurotransmitter release [29], [30]. Gain of function studies demonstrated that tomosyn-1 is responsible for inhibiting exocytosis of dense core granules in primary adrenal chromaffin cells [27], PC12 cells [25], and pancreatic β-cells [15]. Moreover, in vitro biochemical evidence further supports the conclusion that tomosyn-1 inhibits the formation of the SNARE complex [31], [32].

The Ca^2^-independent inhibitory effects of the tomosyn-1 have been attributed to the VLD. More recently, Yamamoto et al demonstrated that in the presence of Ca^2+^, tomosyn-1, via the N-terminal WD40 domain, binds to synaptotagmin and inhibits SNARE complex-mediated neurotransmitter release [26], [30], [33], [34]. Together, the evidence is accumulating for tomosyn-1 as a negative regulator of exocytosis in both the stimulated and unstimulated states.

Insulin resistant animals compensate for their insulin resistance and maintain normal glucose levels by increasing insulin secretion. Our studies show that mutations in tomosyn-2 that increase its inhibitory activity can create a bottleneck and in the presence of obesity-induced insulin resistance, tip the balance towards T2D. However, it is also possible that tomosyn-2 plays an important role in regulating insulin secretion during daily starve/feed cycles by preventing inappropriate insulin secretion during fasting.

Tomosyn-2 may regulate exocytosis by modulating the formation of the SNARE complex in tissues other than islets. We observed significant tomosyn-2 expression in brain, cerebellum, islets, kidney, liver, and gastrocnemius (Figure 7). Therefore it is possible that tomosyn-2, like tomosyn-1, may have an important regulatory role in tissues where regulation of the SNARE complex can be limiting for an important transport process; e.g. insulin-mediated GLUT4 translocation in adipocytes [35] and transport of LDL-derived cholesterol from the trans-Golgi network to the endoplasmic reticulum in hepatocytes [36]. Thus, this tomosyn-2 could be playing a critical role in regulating vesicle trafficking in other tissues.

In summary, we have identified tomosyn-2 as a gene underlying a T2D susceptibility QTL on Chr 16. We show that tomosyn-2 is a negative regulator of insulin secretion. We identified a SNP in tomosyn-2 that affects the stability of the protein and thus suggest a molecular mechanism by which allelic variation in this gene increases diabetes susceptibility. Future studies will focus on the pathways that link nutrient sensing with the role of tomosyn-2 in the regulation of insulin secretion.

## Materials and Methods

### Materials

The enzymatic glucose reagent was purchased from Thermo Scientific. Insulin in lean mice was measured using a radioimmunoassy kit from Linco Research (St. Charles, MO). In *Lep^ob/ob^* mice, insulin was measured with an in-house ELISA using an anti-insulin antibody from Fitzgerald Industries (Acton, MA). The mouse anti-myc antibody and Z-Leu-Leu-Leu-al (MG132) were purchased from Sigma-Aldrich, USA. The mouse secondary antibodies were purchase from Cell Signaling Technology (Boston, MA). Glutathione 4B Sepharose beads were purchased from GE Healthcare, USA.

### Animals and breeding strategy

The C57BL/6 (B6) and BTBR T^+^ tf (BTBR) mice were intercrossed and were crossed to generate F1 mice. The B6.16^B6^ and B6.16^BT^ mice were created by backcrossing the F1 mice to B6 using microsatellite markers to select for BTBR (or B6 in the case of B6.16^B6^) homozygosity on mouse chromosome 16 in an otherwise B6 background. The B6.16^BT^ mice were further backcrossed to B6 with marker assisted selection to create congenic strains. Further identification of the B6/BTBR genetic boundaries were determined by SNP sequencing for some of the congenic strains (listed in Dataset S2. The *Lep^ob^* mutation was introgressed into all strains using *Lep^ob/+^* mice as breeders [37]. All mice were maintained at the Department of Biochemistry, University of Wisconsin-Madison animal care facility on a 12 h dark-light cycle (6 PM to 6 AM). The mice were fed Purina Formulab Chow 5008 and water ad libitum. The mice were kept in accordance with the University of Wisconsin-Madison Research Animals Resource Center and the NIH guidelines for care and use of laboratory animals.

### Plasma measurements

For plasma glucose and insulin measurements, blood was taken from the retro orbital sinus from random fed mice at 8 AM or from fasting mice at 12 PM (fasted at 8 AM). For both *Lep^ob/ob^* and lean mice, glucose was measured via glucose oxidase method (Thermo Scientific). For *Lep^ob/ob^* mice, insulin was measured via ELISA using a matched rat insulin antibody pair (Fitzgerald Industries International Inc.). For lean mice, insulin was measured by Linco Sensitive rat insulin radioimmunoassay.

### Islet isolation and insulin secretion assays

Intact pancreatic islets were isolated from mice using a collagenase digestion procedure [38]. Static insulin secretion assays were performed on preparations consisting of three islets incubated with various secretagogues [38]. For perifusion insulin secretion assays, approximately 100 medium sized islets were washed three times, placed in a sterile Petri dish, and incubated overnight in culture media (RPMI 1640, with 11.1mM glucose, antibiotics and 10% heat inactivated fetal bovine serum). The following day, 50 islets were washed and transferred in 100 µl of Krebs Ringer Buffer (KRB) to the Swinnex filter holder (Millipore). The islets were sandwiched between two layers of Bio-Gel P-2 bead (Bio-Rad) solution (200 mg beads/ml in KRB; bottom layer, 150 µl and top layer, 300 µl). The Swinnex filter holder was attached in-line with a Minipuls 3 pump (Gilson) and a FC 204 Fraction Collector (Gilson). Islets were perifused at the rate of 1ml/min and samples were collected at 30 sec intervals. Islet insulin content and secretion were determined by ELISA.

### Next generation sequencing

Tail DNA was extracted from B6, BTBR, B6.16^BT36–38^, B6.16^BT24–37^and B6.16^BT24–38^ mice using the QIAGEN Puregene Core Kit. RNA baits were designed using Agilent eArray and used for Agilent SureSelect Target Enrichment to capture sequence from a 35.35 to 38.65 Mb region on mouse chromosome 16. Target enrichment was followed by DNA amplification and confirmation of enrichment using SNP sequencing inside and outside of the target region. DNA was sequenced by Next Generation Sequencing using an Illumina GA IIx sequencer at the University of Wisconsin-Madison Biotechnology Center. CLC Genomics 4.0.3 Software was used to identify SNPs and DIPs between B6 and BTBR sequence. For some SNPs and DIPs an additional visual base calling confirmation step was used to test the accuracy of the software (listed in Dataset S1).

### Isolation and quantitation of total RNA

RNA from islets, kidney, and liver was extracted using the QIAGEN RNeasy Plus Kit. RNA from epididymal fat pads, brain, cerebellum, and adrenal glands was extracted using QIAGEN RNeasy Lipid Kit. RNA from heart, soleus, gastrocnemius and quadriceps was extracted using QIAGEN RNeasy Fibrous Tissue Kit. Following extraction, RNA was used for cDNA synthesis (Applied Biosystems). The mRNA abundance was determined by quantitative PCR using FastStart SYBR Green (Roche) and gene expression was represented by comparative ΔCT method.

### Plasmids

MMLV-based retroviral vector (RVV, 3051) (gift from Dr. Bill Sugden, University of Wisconsin, Madison) containing a MCS-IRES GFP was used to generate b-tomosyn-2-RVV construct for expression studies. The pcDNA3-m-tomosyn-1, pCR-Script-xb, -b, -m, and s-tomosyn-2 constructs were generously provided by Dr. Alexander Groffen, Virije Universiteit, Netherlands. We corrected a mutation (AG) at nucleotide 3245 of the b-Tomosyn-2 cDNA. The tomosyn-1 or tomosyn-2 cDNA from these vectors were used for subsequent subcloning. The b-tomosyn-2-RVV construct was generated by subcloning the b-tomosyn-2 cDNA with 5′-BspDI and 3′-NotI overhangs into the compatible 5′-BstBI and 3′-NotI ends of the RVV vector. For binding studies, the tomosyn-2-pcDNA/TO/myc-His was generated by subcloning a PCR-amplified tomosyn-2 cDNA in to 5′-BamHI and 3′-XhoI sites of the pcDNA4/TO/myc-His C vector (Invitrogen). The following primers that were used to amplify tomosyn-2 cDNA with the restriction sites for cloning, a partial KOZAK, and a 3′-precision protease cleavage site are: forward (5′-TTAAAGGATCCGCCACCATGAAGAAGTTTAATTTCCG) and reverse (5′-ATATCTCGAGGGGCCCCTGGAACAGAACTTCCAGGAACTGGTACCACTTCTTATCCT). Similar subcloning strategy was used for generating m-tomosyn-1-pcDNA/TO/myc-His construct. The primers used are as follows: forward (5′-CGAGACCGGATCCGCCACCATGAGGAAATTCAACATC) and reverse (5′-ATATCTCGAGCCCCTGGAACAGAACTTCCAGGAACTGGTACCACTTCTTATCTTTG) primes. The pGEX-4T1-syntaxin-4 construct encoding soluble GST-syntaxin-4 (1-273) fusion protein was generated as previously described [39]. The pGEX-2T1-syntaxin construct (1-265) was a generous gift from Dr. Tom Martin, University of Wisconsin, Madison. All constructs were verified by sequencing.

### Cell culture, transient transfection, and insulin secretion measurements

The glucose responsive rat β-cell line, INS1 (832/13, a gift from Dr. Chris Newgard, Duke University) was cultured in RPMI 1640 medium containing 11 mM glucose supplemented with 10% heat inactivated fetal bovine serum, 2 mM L-glutamine, 1 mM sodium pyruvate, 10 mM HEPES, 100 Units/ml of antibiotic-antimycotic, and 50 µM β-mercaptoethanol. Approximately 100,000 cells/well were plated in a 96-well plate. The following day, INS1 (832/13) cells at 80–90% confluency were transfected with 0.4 µg of plasmid DNA using Lipofectamine 2000 (Invitrogen). After 36 h of incubation, cells were washed once with 200 µl and incubated for 2 h in 100 µl of modified Krebs-Henseleit Ringer bicarbonate buffer (KRB: 118.41 mM NaCl, 4.69 mM KCl, 1.18 mM MgSO_4_, 1.18 mM KH2PO4, 25 mM NaHCO3, 20 mM HEPES, 2.52 mM CaCl2, pH 7.4, and 0.2% BSA) containing 1.5 mM glucose. After 2 h, cells were stimulated for 2 h in 100 µl of KRB buffer containing 7 mM glucose + 3 mM 8-bromo-cAMP. The incubation buffer was collected to determine the amount of insulin secreted under varying conditions. The cells were lysed (lysis buffer: 1 M Tris-HCl, pH 8.0, 1 M NaCl, 0.5 M NaF, 200 mM Na_3_VO_4,_ 2% NP-40, and protease inhibitor cocktail tablet (Roche)) to determine insulin content. The percent fractional insulin secretion was calculated as the amount of insulin secreted divided by total insulin content. Insulin was determined using ELISA.

The human embryonic kidney 293T cells (HEK293T) were cultured in Dulbecco's modified Eagle's medium (DMEM) containing 25 mM glucose were supplemented with 10% fetal bovine serum, 0.1 mM nonessential amino acid, 6 mM L-glutamine, 1 mM sodium pyruvate, 100 units/ml of penicillin, 100 units/ml of streptomycin, and 500 µg/ml of geneticin. HEK293T cells at 70–80% in 100 mm tissue culture dishes were transfect with plasmid DNA constructs using 40 µl of 1 mg/ml polyethylenimine. Following day, cells were lysed (lysis buffer: 20 mM Tris-HCl (pH 7.5). 150 mM NaCl, 1 mM Na_2_EDTA, 1mM EGTA, 1% Triton, 2.5 mM sodium pyrophosphate, 1 mM β-glycerophosphate, 1 mM Na_3_VO_4_, 1 mM PMSF, and protease inhibitor cocktail tablet (Roche)) and total protein lysates were prepared and the immunoblot was performed as described [40]. For protein stability, 16 h post transfection, cells were treated with or without 100 µM MG132 for 6 h. After 6 h, cells were lysed and whole cell lysates were prepared.

### Recombinant proteins, in vitro binding assays, and immunobloting

Recombinant proteins encoding GST or GST-fusion proteins with the cytoplasmic domain of syntaxin-1A and syntaxin-4 were expressed in *E.Coli* strain BL21 (DE3) and were purified using glutathione-affinity chromatography [41]. The concentration and the purity of the fusion proteins were assessed by SDS-PAGE followed by Coomassie-blue staining against BSA standards. The binding studies were preformed by incubating 10 µg of recombinant proteins with 25 µl of 100% Glutathione-Sepharose 4B beads (Amersham Biosciences) with 1 mg of HEK293T lysate overexpressing tomosyn-1 or isoforms of tomosyn-2 (xb, b, m, and s) in 1% Triton-lysis buffer for 2 h at 4°C. After 2 h, the complexes were washed three times with Triton lysis buffer and was eluted in Western loading buffer. The denatured samples were subjected to 10% SDS-PAGE gels followed by transfer to PVDF membrane for immunoblotting. The immunobloting was performed using a standard protocol [40].

### Statistical analysis

Data was expressed as means ± standard error of means. The statistical comparisons were made using Student's *t* test at *p*<0.05.

## Supporting Information

Dataset S1 SNPs and DIPs in QTL Region. Single nucleotide polymorphisms and deletion/insertion polymorphisms (DIPs) in the 36–38.5 Mb region underlying the chromosome 16 QTL were determined by Next Generation sequencing. Using the CLC Genomics 4.0.3 software, for each strain (B6, BTBR, B6.16^BT36–38^, B6.16^BT24–^37, and B6.16^BT24–38^), an algorithm generated a list of SNPs/DIPs using published C57BL/6J sequence as the reference. The algorithm generated a frequency of the SNP/DIP call and only those above 90% were chosen. From there, we sorted the SNPs/DIPs for each strain, and only chose those SNPs/DIPs that were confirmed in at least 3 strains (either one allele for B6 and two for BTBR or vice versa). They also had to agree with what we already knew from our marker and SNP typing (see Supplemental File S1). At that point, to test the validity of the algorithm-generated list, for those that we call “confirmed” by “manual base reading”, we went back to the 4x10^6^ bp sequence that was generated from each strain and confirmed by looking at the numerous reads at each bp call, that this was in fact a real SNP/DIP. We were unable to do this for all SNPs due to time and money constraints, but the accuracy of the algorithm was very good once we placed the above thresholds on the data. The three coding SNPs were not de novo discoveries (although they are not listed in all SNP databases) and were also confirmed via Sanger sequencing in our strains. We cannot say how many were de novo discoveries because the SNP data available for BTBR is very sparse and we have found it to be inaccurate. Previous to embarking on this large-scale sequencing, we used Sanger sequencing to test over 50 published B6 versus BTBR SNPs from the JAX database and found that they were not different between our B6 and BTBR strains; hence the reason for us to do the large-scale sequencing. The DIPs that were discovered were most likely de novo discoveries, because we are unaware that such data exists for DIPs between mouse strains. We did confirm one of these DIPs (DIP37.7) to further define the boundary of strain B6.16^BTBR37–55^. Included in this file are boundaries of the genes in the region and all SNPs & DIPs that satisfied the criteria described above. Those SNPs or DIPs that have base calls for each strain in columns F through J were subject to the above “manual base reading”.
(XLS)

Dataset S2 Tomosyn-2 SNPs. Listed are those SNPs/DIPs that were located upstream and downstream of the tomosyn-2 gene. We identified 1 non-synonymous coding SNP and 8 intronic SNPs in tomosyn-2. Three of the intronic SNPs found are not listed in the MGI database but are listed in UCSC Genome Browser (rs4173887, rs4173899 and rs46721065). We were unable to confirm 2 SNPs listed in MGI and UCSC (rs4173886 and rs4173895). According to published data on the UTR boundaries for tomosyn-2, there were no SNPs found in either 3′ and 5′ UTR. In columns G through K are the base calls for the SNPs/DIPs that were subject to the above “manual base reading”.
(XLS)

Dataset S3 SNP map for chromosome 16. Listed are the microsatellite markers, SNPs and DIPs used to identify the chromosome 16 B6/BTBR boundaries of our congenic mouse strains. Only those with a B6 or BT call were tested at each marker, SNP or DIP. The rest of the region was inferred from flanking markers.
(XLS)

Dataset S4 Gap data. Within our 36–38.5 Mb region on chromosome 16 there were 30531 bp of gap sequence (representing ∼ 1.2% of the region) where there was not sufficient sequence for both B6 and BTBR. This will be an overestimate of actual gaps, due to the fact that the sequence from other strains compared to either B6 or BTBR could still be used to determine SNPs/DIPs with our criteria. Listed are the bp boundaries of the missing B6 and BTBR sequence.
(XLS)

Table S1 Strain distribution of S912L SNP. Non-synonymous coding SNPs identified within Stxbp5l from mouse strains sequenced at the Sanger Institute. Reference sequence corresponds to C57BL/6NJ. -, indicates no change. Data obtained from http://www.sanger.ac.uk/cgi-bin/modelorgs/mousegenomes/snps.pl.
(DOCX)
