# Supplementary material for: Positional Cloning of a Type 2 Diabetes Quantitative Trait Locus; Tomosyn-2, a Negative Regulator of Insulin Secretion
Source: PLoS Genet. 2011 Oct 6;7(10):e1002323. doi: 10.1371/journal.pgen.1002323 (PMC3188574; doi:10.1371/journal.pgen.1002323)
Supplement: Table S1 — Strain distribution of S912L SNP. Non-synonymous coding SNPs identified within Stxbp5l from mouse strains sequenced at the Sanger Institute. Reference sequence corresponds to C57BL/6NJ. -, indicates no change. Data obtained from http://www.sanger.ac.uk/cgi-bin/modelorgs/mousegenomes/snps.pl. (DOCX) [file pgen.1002323.s005.docx]

| **Gene** | **Chromosome** | **Position (bp)** | **Reference** | **129P2** | **129S1/SvlmJ** | **129S5** | **A/J** | **AKR/J** | **BALB/cJ** | **C3H/HeJ** | **C57BL/6NJ** | **CAST/EiJ** | **CBA/J** | **DBA/2J** | **LP/J** | **NOD/ShiLtJ** | **NZO/HILtJ** | **PWK/PhJ** | **Spretus/EiJ** | **WSB/EiJ** |
| --- | --- | --- | --- | --- | --- | --- | --- | --- | --- | --- | --- | --- | --- | --- | --- | --- | --- | --- | --- | --- |
| **Stxbp5l** | **16** | **37134466** | **G** | - | - | - | **A** | **A** | **A** | **A** | - | - | **A** | **A** | **A** | **A** | **A** | - | - | - |
|  | **16** | **37140044** | **C** | - | - | - | - | - | - | - | - | **T** | - | - | - | - | - | - | - | **T** |
|  | **16** | **37208147** | **C** | - | - | - | - | - | - | - | - | - | - | - | - | - | - | - | **T** | - |
|  | **16** | **37330002** | **C** | - | - | - | - | - | - | - | - | - | - | - | - | - | - | **T** | - | - |
|  | **16** | **37383662** | **G** | - | - | - | - | - | - | - | - | - | - | - | - | - | - | - | **A** | - |
